# Visit‐to‐visit office blood pressure variability combined with Framingham risk score to predict all‐cause mortality: A post hoc analysis of the systolic blood pressure intervention trial

**DOI:** 10.1111/jch.14314

**Published:** 2021-07-03

**Authors:** Yi Cheng, Jian Li, Xinping Ren, Dan Wang, Yulin Yang, Ya Miao, Chang‐Sheng Sheng, Jingyan Tian

**Affiliations:** ^1^ Department of Cardiovascular Medicine Center for Epidemiological Studies and Clinical Trials and Center for Vascular Evaluation Shanghai Key Lab of Hypertension Shanghai Institute of Hypertension Ruijin Hospital Shanghai Jiaotong University School of Medicine Shanghai China; ^2^ Clinical Research Center Ruijin Hospital Shanghai Jiaotong University School of Medicine Shanghai China; ^3^ Department of Ultrasound Ruijin Hospital Shanghai Jiao Tong University School of Medicine Shanghai China; ^4^ State Key Laboratory of Medical Genomics Clinical Trial Center Department of Endocrinology and Metabolism Shanghai Institute of Endocrine and Metabolic Diseases Ruijin Hospital Shanghai Jiaotong University School of Medicine Shanghai China

**Keywords:** Framingham risk score, mortality, the systolic blood pressure intervention trial, visit‐to‐visit blood pressure variability

## Abstract

We aim to determine if visit‐to‐visit blood pressure variability (BPV) adds prognostic value for all‐cause mortality independently of the Framingham risk score (FRS) in the systolic blood pressure intervention trial (SPRINT). We defined BPV as variability independent of the mean (VIM) and the difference of maximum minus minimum (MMD) of the systolic blood pressure (SBP). Multivariable Cox proportional hazards models were used to estimate the hazard ratio (HR) and 95% confidence interval (CI). Based on FRS stratification, there were 1035, 2911, and 4050 participants in the low‐, intermediate‐, and high‐risk groups, respectively. During the trial, 230 deaths occurred since the 12th month with an average follow‐up of 2.5 years. In continuous analysis, 1‐SD increase of SBP VIM and MMD were significantly associated with all‐cause mortality (HR 1.18, 95% CI 1.05–1.32, *p* = .005; and HR 1.21, 95% CI 1.09–1.35, *p* < .001, respectively). In category analysis, the highest quintile of BPV compared with the lowest quintile had significantly higher risk of all‐cause mortality. Cross‐tabulation analysis showed that the 3rd tertile of SBP VIM in the high‐risk group had the highest HR of all‐cause mortality in total population (HR 4.99; 95% CI 1.57–15.90; *p* = .007), as well as in intensive‐therapy group (HR 7.48; 95% CI 1.01–55.45; *p* = .05) analyzed separately. Cross‐tabulation analysis of SBP MMD had the same pattern as VIM showed above. In conclusion, visit‐to‐visit BPV was an independent predictor of all‐cause mortality, when accounting for conventional risk factors or FRS. BPV combined with FRS conferred an increased risk for all‐cause mortality in the SPRINT trial.

## 1 INTRODUCTION

Hypertension is a highly prevalent chronic disease around the world, and remains the most common risk factor for cardiovascular diseases (CVD) and mortality.^1^ In the management of hypertension, several current guidelines recommend that other major risk factors (such as sex, age, diabetes, smoking, and cholesterol) should also be considered together.^2^, ^3^ Scoring equations such as Framingham risk score (FRS) and atherosclerotic cardiovascular disease (ASCVD) risk score, using abovementioned cardiovascular risk factors, have been developed in the risk evaluation^4^, ^5^ and antihypertensive treatment in hypertensive patients.^2^, ^3^ In the systolic blood pressure intervention trial (SPRINT), a Framingham 10‐year risk of general CVD exceeding 15% was used to identify increased CVD risk.^6^

Besides general cardiovascular risk factors, visit‐to‐visit office blood pressure variability (BPV) has also become a hot topic in the management of hypertension. Since Rothwell and colleagues suggested that visit‐to‐visit BPV was an independent predictor of stroke,^7^ several studies have confirmed BPV as an independent predictor of cardiovascular events, stroke, myocardial infarction, and cardiovascular mortality.^8^, ^9^, ^10^, ^11^, ^12^ In a recent post hoc analysis of SPRINT, BPV was reported as a significant predictor of all‐cause mortality independent of general risk factors.^13^

The objective of our study was to determine if the BPV provides information on the risk of all‐cause mortality independently of the Framingham risk score and can improve risk prediction, using data from the SPRINT.

## 2 METHODS

### 2.1 Study population

SPRINT was a multicenter randomized, controlled study cohort, designed to find out whether intensive BP lowering target (systolic target <120 mmHg) could reduce CVD risks, compared with standard target (systolic target <140 mmHg).^7^, ^14^ Overall, SPRINT trial collected 9361 hypertensive participants with increased risks of CVD, who were at least 50 years old with SBP ≥130 mmHg, evidence of CVD, chronic kidney disease (CKD), or a 10‐year FRS score ≥15%. Participants were randomly assigned to standard‐ or intensive‐therapy groups. At each visit, the average BP was recorded based on three BP measurements with the use of an automated measurement system (Model 907, Omron Healthcare).^15^ Clinic visits were designed occurring at baseline and 1‐, 2‐, and 3‐month, and then turned to once every 3 months, up to 6 years. However, based on significantly lower rate of the primary outcomes in intensive‐therapy arm, the trial ceased at 4 years and 9 months (median follow‐up, 3.26 years). The outcome of all‐cause death was defined as confirmed deaths of any causes.

### 2.2 Risk stratification

Framingham risk scores were calculated for participants without baseline CVD history based on the following risk factors: age, smoking status, systolic blood pressure, high‐density lipoprotein (HDL), total cholesterol (TC), and diabetes.^4^ For further analysis, subjects were then stratified into three subgroups according to their FRS and baseline CVD history: low (FRS <10%), intermediate (10% ≤ FRS < 20%), and high (FRS ≥20% or having baseline CVD history) risk groups.

### 2.3 Visit‐to‐visit blood pressure variability

Since SPRINT trial focused on different systolic blood pressure (SBP) lowering targets, our study of BPV also concentrated on SBP. In order to reach new BP target, most participants in intensive arm had to change their previous antihypertensive regimen, which brought additional BP fluctuation. After 3‐month visit, BP in both groups reached relatively stable plateau (Table S1 in the Supplement). Therefore, we chose to use four BP recordings of 3‐, 6‐, 9‐, and 12‐month visits to avoid the inference of medication at the beginning of the trial. In a sensitivity analysis, we also considered five BP measurements taken during the 3‐, 6‐, 9‐, and 12‐month study visits, and six BP measurements taken during the 3‐, 6‐, 9‐, 12‐, 15‐, and 18‐month study visits.

We defined BPV as variability independent of the mean (VIM) and the difference of maximum minus minimum (MMD). VIM, a new index which can diminish the tight correlation between the coefficient of variation and mean.^16^ VIM is calculated as the SD divided by the mean to the power x and multiplied by the population mean to the power x, with x derived from curve fitting.^17^ MMD is calculated as maximum minus minimum SBP of 3‐ to 12‐month visits, which might potentially be another indicator of BPV.^15^, ^18^, ^19^

For the current analysis, we excluded 1331 participants with any one of the four BP records missed, and 34 without FRS.

### 2.4 Statistical analysis

For database management and statistical analysis, we used SAS software version 9.4 (SAS Institute Inc., Cary, NC). Baseline characteristics were described as a whole and also compared between BP lowering targets and risk stratifications (FRS <10%, 10%–19%, and ≥20% or having baseline CVD history), respectively. Normal continuous variables were presented as mean±SD, while categorical variables were n (percentage). Significance was a 2‐tailed α‐level of ≤0.05. Means and proportions were compared using the large‐sample z test and the χ2 statistic, respectively.

The correlation between BPV (VIM and MMD) and all‐cause mortality was performed by Cox proportional hazards regression as continuous and category variables. Kaplan–Meier survival probabilities were estimated according to VIM tertiles of SBP stratified by FRS categories, and differences were analyzed by the log‐rank test. Cox proportional hazards regression was also performed to detect the association between BPV and all‐cause mortality based on FRS stratification. There were two multivariable adjusted models conducted: (1) adjustment for BP randomized therapy and risk stratification; (2) further adjustment of plasma glucose, chronic kidney disease, average number of antihypertensive agents, and the use of statin and aspirin. The prediction value of BPV based on risk stratifications was investigated by Cox proportional hazards regression while BPV was categorized as tertiles. For each Cox proportional hazards regression, Wald test was then conducted to determine the statistical significance.

## 3 RESULTS

### 3.1 Characteristics of study participants

Of the original 9361 participants enrolled in SPRINT, 7996 (3995 standard group; 4001 intensive group) met the inclusion and exclusion criteria and were included in the current analysis. Mean age was 68.0 years, and 34.8% were women. Key baseline characteristics were similar in the standard and intensive therapy groups (Table 1). Mean SBP and DBP levels were relatively stable from the 3rd month to the end of study after a rapidly falling off from baseline to the 2nd month especially in the intensive‐therapy group (Table S1 in the Supplement).

**TABLE 1:** Baseline characteristics of the study population

		Therapy status		Risk levels		
	All participants (n = 7996)	Standard (n = 3995)	Intensive (n = 4001)	Low‐risk (n = 1035)	Intermediate‐risk (n = 2911)	High‐risk (n = 4050)
**At baseline**						
Age (years)	68.0 ± 9.3	67.9 ± 9.3	68.0 ± 9.3	62.4 ± 7.9	65.8 ± 8.6	70.9 ± 9.1***
Female	2780 (34.8)	1367 (34.2)	1413 (35.3)	845 (81.6)	1311 (45.0)	624 (15.4)***
Black race	2411 (30.2)	1225 (30.7)	1186 (29.6)	513 (49.6)	1010 (34.7)	888(21.9)***
BMI (kg/m^2^)	29.9 ± 5.7	29.8 ± 5.6	30.0 ± 5.8	31.5 ± 6.7	30.3 ± 5.9	29.2 ± 5.2***
Smoking status						
Never smoked	3547 (44.4)	1775 (44.4)	1772 (44.3)	613 (59.2)	1445 (49.6)	1489 (36.8)***
Former smoker	3457 (43.2)	1739 (43.5)	1718 (42.9)	347 (33.5)	1220 (41.9)	1890 (46.7)***
Current smoker	992 (12.4)	481 (12.0)	511 (12.8)	75 (7.2)	246 (8.5)	671 (16.6)***
SBP (mmHg)	139.7 ± 15.5	139.7 ± 15.3	139.6 ± 15.6	128.3 ± 12.4	138.4 ± 13.4	143.5 ± 16.0***
DBP (mmHg)	78.1 ± 11.9	78.0 ± 12.0	78.2 ± 11.8	76.4 ± 10.9	79.0 ± 11.2	77.9± 12.6***
No drug use	769 (9.6)	397 (9.9)	372 (9.3)	14 (1.4)	165 (5.7)	590 (14.6)***
Number of drugs	1.8 ± 1.0	1.8 ± 1.0	1.9 ± 1.0	2.0 ± 0.9	1.9 ± 1.0	1.7 ± 1.1***
Statin	3539 (44.3)	1792(44.9)	1747 (43.7)	393 (38.0)	1181 (40.6)	1966 (48.5)***
Aspirin	4153 (51.9)	2038 (51.0)	2115 (52.9)	408 (39.4)	1342(46.1)	2403 (59.3)***
History of CKD	2236 (28.0)	1103 (27.6)	1133 (28.3)	274 (26.5)	710 (24.4)	1252 (30.9)***
eGFR (ml/min/1.73m^2^)	71.8 ± 20.4	71.8 ± 20.2	71.8 ± 20.5	73.8 ± 22.2	73.5 ± 20.1	70.1 ± 19.9***
Serum creatinine (mg/dL)	1.1 ± 0.3	1.1 ± 0.3	1.1 ± 0.3	1.0 ± 0.3	1.0 ± 0.3	1.1 ± 0.3***
Fasting total cholesterol (mg/dL)	189.6 ± 40.8	189.7 ± 40.6	189.4 ± 40.9	190.1 ± 36.5	193.1 ± 39.0	186.8 ± 42.8***
Fasting HDL (mg/dL)	52.7 ± 14.4	52.7 ± 14.5	52.7 ± 14.3	59.6 ± 16.6	54.8 ± 14.6	49.5± 12.7***
Fasting triglycerides (mg/dL)	126.1 ± 87.4	126.9 ± 93.4	125.3 ± 81.1	105.7 ± 59.5	120.5 ± 67.8	135.3 ± 103.3***
Fasting plasma glucose (mmol/l)	99.0 ± 13.5	98.9 ± 13.3	99.1 ± 13.7	97.0 ± 14.6	99.0 ± 13.7	99.6 ± 13.0***
**During follow up**						
SBP mean (mmHg)	128.2 ± 10.9	134.9 ± 8.2	121.6 ± 9.0***	125.5± 11.1	128.1 ± 10.6	129.0 ± 10.9***
SBP SD (mmHg)	10.1 ± 5.9	10.5 ± 6.0	9.6 ± 5.8***	9.8 ± 5.8	9.8 ± 5.6	10.3 ± 6.0**
DBP mean (mmHg)	71.9 ± 9.6	75.3 ± 9.4	68.5 ± 8.5***	74.3 ± 8.9	73.4 ± 9.2	70.2 ± 9.7***
DBP SD (mmHg)	6.1 ± 3.2	6.3 ± 3.2	5.9 ± 3.1***	6.1 ± 3.2	6.0 ± 3.2	6.1 ± 3.2
SBP VIM (unit)	10.0 ± 5.6	10.5 ± 5.8	9.5 ± 5.3***	10.2 ± 5.7	9.8 ± 5.4	10.1 ± 5.7*
SBP MMD (mmHg)	22.4 ± 13.1	23.4 ± 13.3	21.4 ± 12.9***	21.7 ± 12.9	21.8 ± 12.6	22.9 ± 13.5***

Abbreviations: ASCVD, atherosclerotic cardiovascular diseases; BMI, body mass index; CKD, chronic kidney diseases; CVD, cardiovascular diseases; DBP, diastolic blood pressure; eGFR, estimated glomerular filtration rate; HDL, high‐density lipoprotein cholesterol; MMD, the difference of maximum minus minimum; SBP, systolic blood pressure; VIM, variability independent of the mean.

Values are mean ± SD or number of subjects (%).

*p < .05; **p < .01; ***p < .001.

Compared with the standard‐therapy group, the intensive‐therapy group had significantly lower follow‐up mean SBP levels in the reduced subjects in our analysis (128.2 vs. 134.9 mmHg, *p* < .001) and had significantly (*p* < .001) lower SBP variability indices, including SD (10.1% vs. 10.5%), VIM (9.5U vs. 10.5U), and MMD (23.4 mmHg vs. 21.4 mmHg). Similar results were also observed in DBP levels and variability.

Based on risk stratification, there were 1035, 2911, and 4050 participants in the low‐, intermediate‐, and high‐risk groups, respectively. Table 1 summarized the characteristics of the participants by FRS stratification. For baseline characteristics, the participants in high‐risk group were significantly older and less frequently in women and black race, but more frequently in current smokers, who had lower BMI and eGFR, total and HDL cholesterol with higher triglycerides, and higher rate of CKD history. The participants in high‐risk group had significantly higher mean BP levels and SD of SBP during follow‐up. SBP VIM had significant difference between risk stratification though the difference was minor, and SBP MMD was significant higher in high‐risk group (Table 1).

Results of five BP measurements taken during the 3‐, 6‐, 9‐, and 12‐month study visits, and six BP measurements taken during the 3‐, 6‐, 9‐, 12‐, 15‐, and 18‐month study visits were available in Tables S2 and S3 in the Supplement.

### 3.2 BPV and all‐cause mortality

During the trial, 230 deaths (138 in standard arm; 92 in intensive arm) occurred since the 12th month with an average follow‐up of 2.5 years. Compared with intensive‐therapy groups, participants randomized into standard‐therapy arms had higher incidence of all‐cause mortality (10.3 vs. 6.9 cases/1000 person‐years).

In continuous analysis with fully adjustment, a 1‐SD increase of SBP VIM was significantly associated with all‐cause mortality (HR 1.18, 95% CI 1.05–1.32, *p* = .005) in total population and in standard‐therapy group (HR 1.19; 95% CI 1.04–1.36; *p* = .01), but not in intensive‐therapy group. MMD also had significant association with all‐cause mortality (HR 1.21, 95% CI 1.09–1.35, *p* < .001) overall and in different BP treatments (standard: HR 1.17, 95% CI 1.01–1.35, *p* = .03; intensive: HR 1.30, 95% CI 1.09–1.53, *p* = .003) (Table 2).

**TABLE 2:** Hazard ratio of SBP variability for all‐cause mortality (four BP measurements)

	Overall (n = 7996)		Standard therapy (n = 3995)		Intensive therapy (n = 4001)	
	Model 1	Model 2	Model 1	Model 2	Model 1	Model 2
**SBP VIM**						
**Continuous**						
+5.6 U	1.21(1.08‐1.36)***	1.18(1.05‐1.32)**	1.25(1.08‐1.43)**	1.19(1.04‐1.36)*	1.15(0.95‐1.41)	1.15(0.94‐1.40)
**Quintiles**						
Q1	Reference	Reference	Reference	Reference	Reference	Reference
Q2	1.24(0.77‐1.98)	1.20(0.75‐1.92)	1.51(0.81‐2.81)	1.45(0.78‐2.71)	0.94(0.45‐1.95)	0.91(0.44‐1.90)
Q3	1.60(1.03‐2.49)*	1.53(0.98‐2.39)	1.74(0.95‐3.16)	1.65(0.91‐3.01)	1.45(0.75‐2.81)	1.42(0.73‐2.76)
Q4	1.48(0.95‐2.31)	1.40(0.90‐2.20)	1.39(0.76‐2.57)	1.32(0.72‐2.44)	1.64(0.85‐3.16)	1.57(0.81‐3.03)
Q5	1.87(1.22‐2.87)**	1.71(1.11‐2.63)*	2.08(1.18‐3.66)*	1.85(1.05‐3.26)*	1.60(0.67‐4.62)	1.54(0.79‐3.02)
**SBP MMD**						
**Continuous**						
+13.1 mmHg	1.26(1.14‐1.40)***	1.21(1.09‐1.35)***	1.23(1.07‐1.40)**	1.17(1.01‐1.35)*	1.31(1.12‐1.55)***	1.30(1.09‐1.53)**
**Quintiles**						
Q1	Reference	Reference	Reference	Reference	Reference	Reference
Q2	1.32(0.78‐2.23)	1.27(0.75‐2.16)	1.30(0.66‐2.55)	1.25(0.63‐2.46)	1.36(0.59‐3.13)	1.31(0.57‐3.03)
Q3	1.72(1.04‐2.84)*	1.63(0.99‐2.71)	1.74(0.92‐3.30)	1.66(0.87‐3.14)	1.66(0.73‐3.75)	1.60(0.70‐3.63)
Q4	1.99(1.23‐3.21)**	1.86(1.15‐3.00)*	1.73(0.93‐3.21)	1.61(0.87‐2.98)	2.47(1.16‐5.27)*	2.34(1.09‐5.02)*
Q5	2.28(1.42‐3.67)***	2.04(1.26‐3.30)**	1.92(1.04‐3.53)*	1.66(0.90‐3.08)	3.00(1.41‐6.37)**	2.84(1.32‐6.08)**

Model 1 with adjustment of randomized group and FRS stratification.

Model 2 further adjusted with history of CKD, fasting glucose, mean number of antihypertensive agents, and statin and aspirin use.

Abbreviations: MMD, the difference of maximum minus minimum; Q1‐5, quintile 1–5; SBP, systolic blood pressure; VIM, variability independent of the mean.

*p < .05; **p < .01; ***p < .001.

In category analysis with similar adjustment, the highest quintile of SBP VIM compared with the lowest quintile had significantly higher risk of all‐cause mortality in total population (HR 1.71; 95% CI 1.11–2.63; *p* = .01) and in standard‐therapy group (HR 1.85; 95% CI 1.05–3.26; *p* = .03), but not in intensive‐therapy group with full adjustment. Highest quintile of SBP MMD had significant prognostic value of death (HR 2.04, 95% CI 1.26–3.30, *p* = .004) overall and in intensive therapy group (HR 2.84, 95% CI 1.32–6.08, *p* = .007) in full model (Table 2).

### 3.3 BPV combined with risk stratification of all‐cause mortality

**FIGURE 1:**
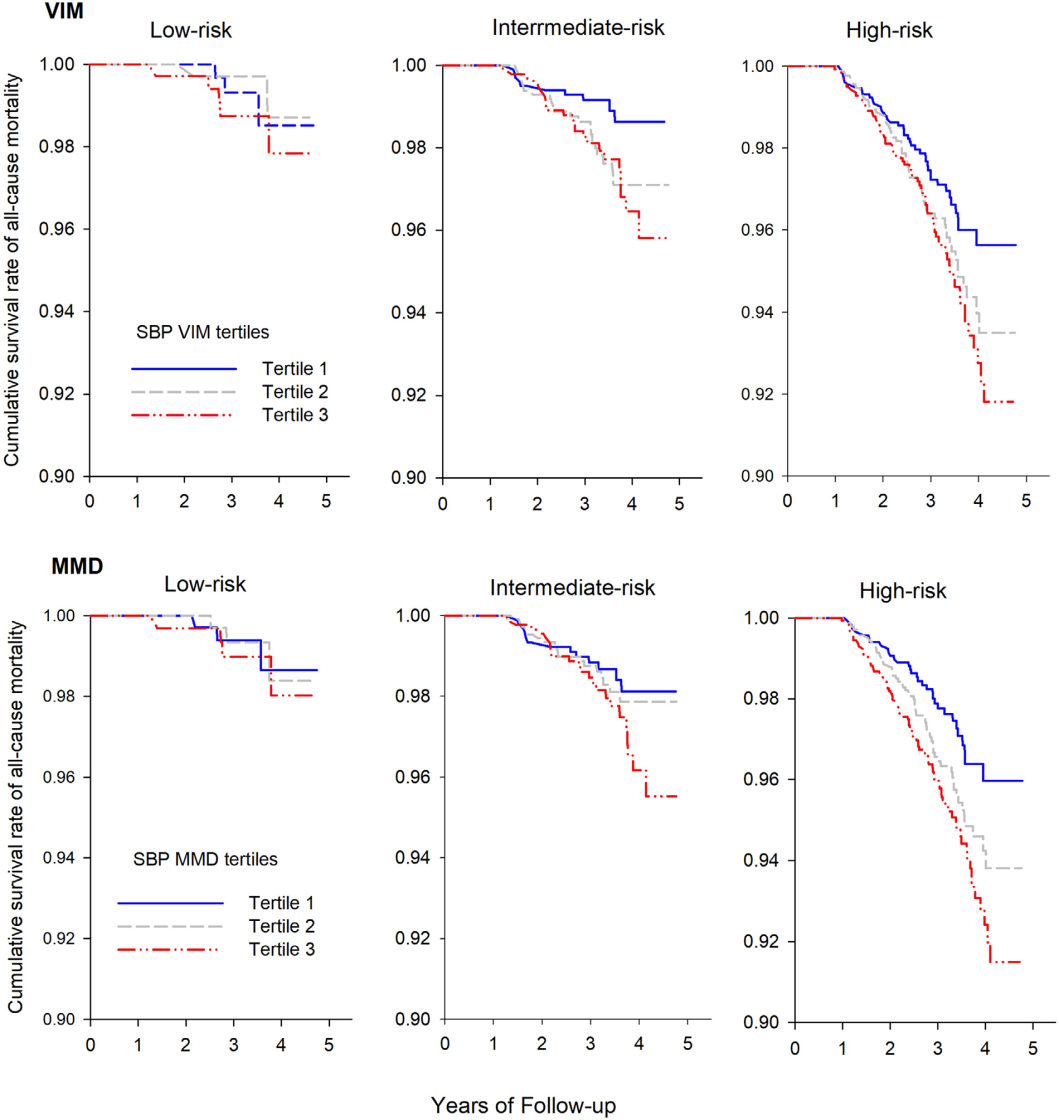
Category analysis on the association between blood pressure variability (SBP VIM and SBP MMD) and all‐cause mortality by different FRS stratification (low‐, intermediate‐, and high risk). Kaplan–Meier survival curve was performed for all‐cause mortality according to the tertiles of SBP VIM and MMD. Abbreviations: FRS, Framingham risk score; MMD, difference of maximum minus minimum; SSB, systolic blood pressure; VIM, variability independent of the mean

The incidence of all‐cause mortality was significantly higher with increased risk levels (FRS <10%, 10%–19%, and ≥20% or having baseline CVD history as low‐, intermediate‐, and high‐risk, respectively). The all‐cause mortality was increased with higher BPV tertiles in all low‐, intermediate‐, and high‐risk groups, and a significant increase was in intermediate‐risk group of VIM and high‐risk group of MMD (*p* = .05 and *p* = .004, respectively. Figure 1).

**FIGURE 2:**
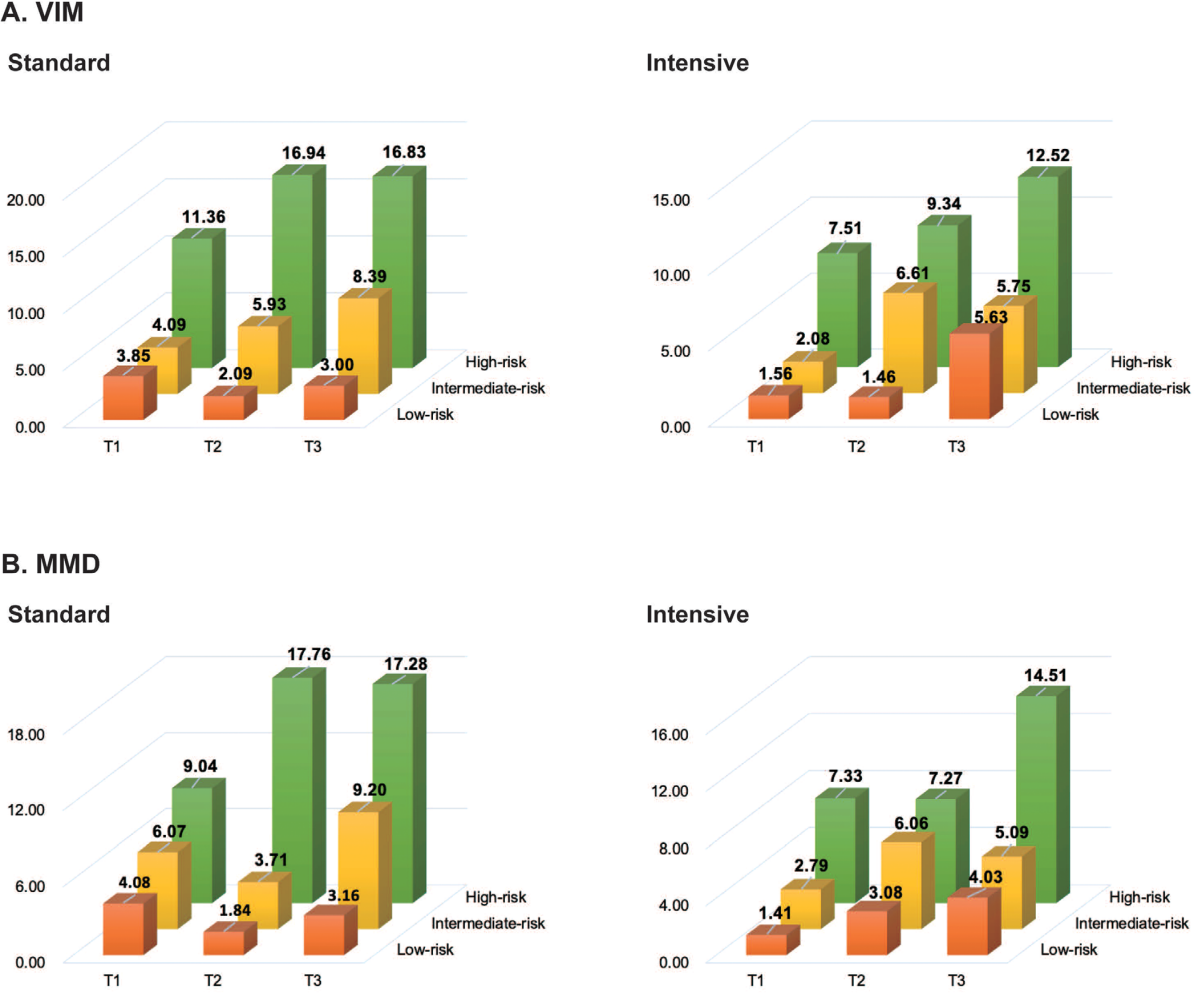
Cross‐tabulation analysis of blood pressure variability (SBP VIM and SBP MMD) and FRS. As the tertiles of BPV and FRS increased, the incidence of all‐cause mortality increased significantly. The third tertile of blood pressure variability combined with the high‐risk category of FRS had the highest incidence of all‐cause mortality. Abbreviations: BPV, blood pressure variability; FRS, Framingham risk score; MMD, difference of maximum minus minimum; SSB, systolic blood pressure; VIM, variability independent of the mean

We further conducted a cross‐tabulation analysis of BPV tertiles and risk stratifications in relation to all‐cause mortality by Cox proportional hazards regression (Table 3 and Figure 2). Overall, as SBP BPV tertiles and risk stratifications increased, so did the HRs of all‐cause mortality. And the 3rd tertile of SBP BPV combined with high‐risk category of FRS had the highest HR of all‐cause mortality in total population (VIM: HR 4.99; 95% CI 1.57–15.90, *p* = .007; MMD: HR 5.45, 95% CI 1.71–17.32, *p* = .004), as well as in the intensive‐therapy group (VIM: HR 7.48; 95% CI 1.01–55.45, *p* = .05; MMD: HR 9.63, 95% CI 1.31–70.92, *p* = .03).

**TABLE 3:** Cross‐tabulation of FRS levels and variability tertiles in relation to all‐cause mortality

Risk stratification	Low‐risk	Intermediate‐risk	High‐risk
**VIM Tertiles** Overall (n = 7996)			
T1	Reference	1.13(0.31‐4.11)	3.40(1.05‐11.00)*
T2	0.67(0.11‐4.04)	2.30(0.69‐7.72)	4.55(1.42‐14.55)*
T3	1.44(0.34‐6.03)	2.41(0.72‐8.05)	4.99(1.57‐15.90)**
Standard therapy (n = 3995)			
T1	Reference	1.07(0.22‐5.31)	2.89(0.68‐12.23)
T2	0.58(0.052‐6.37)	1.58(0.35‐7.22)	4.14(0.99‐17.23)
T3	0.74(0.10‐5.29)	2.03(0.46‐8.89)	3.94(0.95‐16.34)
Intensive therapy (n = 4001)			
T1	Reference	1.31(0.15‐11.71)	4.52(0.60‐34.05)
T2	0.90(0.056‐14.45)	3.96(0.51‐33.71)	5.46(0.73‐40.84)
T3	3.29(0.34‐31.70)	3.27(0.41‐26.22)	7.48(1.01‐55.45)*
**MMD Tertiles** Overall (n = 7996)			
T1	Reference	1.66(0.47‐5.82)	3.06(0.94‐10.02)
T2	0.98(0.20‐4.85)	1.81(0.53‐6.14)	4.50(1.41‐14.40)*
T3	1.27(0.28‐5.68)	2.54(0.76‐8.48)	5.45(1.71‐17.32)**
Standard therapy (n = 3995)			
T1	Reference	1.51(0.32‐7.13)	2.19(0.50‐9.18)
T2	0.47(0.042‐5.14)	0.91(0.19‐4.40)	4.10(0.99‐16.99)
T3	0.75(0.11‐5.31)	2.11(0.48‐9.18)	3.79(0.92‐15.69)
Intensive therapy (n = 4001)			
T1	Reference	1.98(0.23‐16.96)	5.02(0.66‐38.09)
T2	2.11(0.19‐23.37)	4.14(0.53‐32.22)	4.90(0.65‐37.00)
T3	2.75(0.25‐30.47)	3.29(0.40‐26.84)	9.63(1.31‐70.92)*

Models were adjusted with randomized group, history of CKD, glucose, mean number of antihypertensive agents, and statin and aspirin use.

Abbreviations: MMD, max‐min difference; T1‐3, tertile 1–3; SBP, systolic blood pressure; VIM, variation independent of the mean.

*p < .05; **p < .01.

We also calculated the c‐statistic to estimate the incremental prognostic value of the BPV. SBP VIM did not show increasing prognostic value for all‐cause mortality beyond risk stratification (c‐statistic from 0.668–0.677, *p* = .09), but SBP MMD had significant increase of concordance (c‐statistic from 0.668–0.682, *p* = .02).

### 3.4 Sensitivity analysis

We further did similar analysis in participants without baseline CVD history (results were shown in Tables S4 and S5 in the Supplement). SBP VIM and SBP MMD had significant prognostic value of all‐cause mortality as continuous and categorical variables in participants without CVD history. Combined with risk stratification, highest BPV tertile in high‐risk group had significant highest risk of all‐cause mortality (VIM: HR 4.34, 95% CI 1.33–14.13, *p* = .01; MMD: HR 4.78, 95% CI 1.48–15.49, *p* = .009).

## 4 DISCUSSION

In the SPRINT, the prognostic significance of all‐cause mortality for FRS^20^ and visit‐to‐visit BPV^13^ has been previous investigated separately. The current study was the first to investigate the combined effect of these two indices. The key findings can be summarized in two points: (1) Visit‐to‐visit BPV was an independent predictor of all‐cause mortality, when accounting for conventional risk factors or FRS; (2) BPV and FRS were both risk factors of all‐cause mortality, and higher BPV combined with higher FRS conferred an increased risk for all‐cause mortality in all patients as well as in the intensive‐therapy group. Nonetheless, BPV didn't show increasing all‐cause mortality risk beyond FRS. These finding implied that visit‐to‐visit BPV might be a useful marker over and beyond traditional risk factor, and should be taken into account for the cardiovascular risk assessment.

The prognostic significance of BPV has been investigated in clinical trials but remained controversy. In 2010, Rothwell and colleagues published a comprehensive series of analyses from four clinical studies showing strong associations between BPV and stroke and cardiovascular risk.^17^ Since then, a number of studies have evaluated the associations between BPV and outcomes.^9^, ^12^, ^21^, ^22^ The post hoc analysis of ADVANCE trial (Action in Diabetes and Vascular Disease: Preterax and Diamicron MR Controlled Evaluation) indicated that visit‐to‐visit BPV was an independent risk factor of myocardial infarction and cardiovascular death but not stroke in diabetes population. However, other studies showed different results.^8^ Analysis of Systolic Hypertension in Europe (Syst‐Eur) trial and European Lacidipine Study on Atherosclerosis (ELSA) database showed no significant correlation between visit‐to‐visit BPV and cardiovascular events.^23^, ^24^ These conflicting results might be probably because of the inconsistence of study designs and the number of visits used in these analyses. The phenomenon limits the clinical practice of visit‐to‐visit BPV. Therefore, large‐scale and well‐designed cohorts, together with standard calculation methods of visit‐to‐visit BPV, are in urgent need.

The present study was consistent with a prior post hoc analysis in the SPRINT, using four office BP measurements from the 3‐, 6‐, 9‐, and 12‐month study visits, and found that the highest quintile of BPV was associated with all‐cause mortality.^13^ However, we used VIM and MMD as indices of BPV instead. The variability index, VIM, can diminish the tight correlation between the CV and mean while MMD could directly reflect the fluctuation of BP and did not show close correlation with the mean (correlation = 0.25, *p* < .001), which might be more stable and suitable so as to showing significant association even adjusted for other conventional factors or FRS.^25^ Previous reports showed different statistics of BPV might affect a lot. In our study, when using five or six measurements to calculate BPV, VIM, and MMD had different performance. The association between SBP VIM and all‐cause mortality was strengthened, nor was SBP MMD. The probable explanation of this phenomenon might because BP levels reached stable plateau after 1‐year visit, and MMD failed to detect minor variation of SBP.

Our present study clearly showed that BPV combined with FRS had higher risk of all‐cause mortality. Participants in high‐risk group were most vulnerable to BP fluctuation. To some extent, atherosclerosis/arteriosclerosis might explain this phenomenon. Visit‐to‐visit BPV was believed to have tight association with arterial stiffness in various mesures.^26^ Nagai and colleagues found exaggerated visit‐to‐visit BP fluctuations were significant indicators for carotid artery atherosclerosis and stiffness independently of average BP.^18^ According to Okada and colleagues, visit‐to‐visit BPV had significant relationship with pulse wave velocity (PWV) and ankle‐brachial index (ABI) which reflected the degree of arteriosclerosis and atherosclerosis, respectively.^27^ Therefore, patients having higher BPV were more likely to have artery atherosclerosis and stiffness. Our finding verified the cumulative effect of visit‐to‐visit BPV and FRS. High visit‐to‐visit BPV and high FRS could mutually strengthen the prognostic risk of all‐cause mortality.

FRS is a widely‐used score to evaluate the CVD risk in the general population free of CVD, containing age, sex, smoking, antihypertensive treatment, baseline SBP, and cholesterol levels and predicts the CVD risk by stratifying individuals into three risk categories: low (<10% risk of an event in 10 years), intermediate (10%–20%), and high (>20%).^4^ Clinical guidelines recommend FRS, as well as other scoring equations, as a tool for risk assessment in hypertensive patients.^3^ Nonetheless, FRS is often considered the reference standard but has limited accuracy, tending to over‐estimate risk in low risk populations and under‐estimate in high risk populations.^28^ The incorporation of other risk markers, such as metabolic syndrome,^29^ plasma C‐reactive protein (C‐RP),^30^, ^31^ and ABI^32^ has had partial success in improving prediction. Besides abovementioned makers, the variation of BP might also be important factor. BPV related closely to many CVD risk factors^33^, ^34^ which enrolled in the scoring equations of FRS. To the best of our knowledge, the present study was the first to study the combined effect of BPV and FRS for all‐cause mortality, and found higher BPV combined with higher FRS conferred the highest risk for the hard endpoint.

In the present analysis, the BPV showed statistical significance between standard‐ and intensive‐therapy group after removal of BP readings from baseline to the 2‐month visit where the BP sharply went down in intensive‐therapy group. The higher BPV combined with higher FRS conferred an increased risk for all‐cause mortality in the intensive‐therapy but not in the standard‐therapy group. While intensive therapy reduced the risk of all‐cause mortality more, hypertensive individuals with uncontrolled BPV still have excess risk. Novel therapies or drug combinations addressing BPV might further reduce this excess risk not only due to BP levels but also the variability. Studies of the association of BPV and CVD outcomes may help understand mechanistic links between hypertension and CVD and, thus, lead to more efficacious therapy.

Our study should be interpreted within the context of its strengths and limitations. The strengths of our study include that SPRINT is a well‐designed, randomized controlled study, allowing for large subgroups of those with different FRS at baseline. The unattended office BP measurements were carefully ascertained to limit over‐ or underestimation of clinic BP. The assessment of OBPV (visit‐to‐visit office blood pressure variability) started from the 3‐month visit so as to avoid period when medications were most actively titrated.

Our analysis should also consider its limitations. The study of SPRINT trial was designed to investigate the prognosis of different SBP lowering targets, but was not designed particularly for longitudinal assessment of visit‐to‐visit BPV. A well‐designed prospective study with a large sample size should be conducted to assess the BPV and FRS in these populations and validate the findings of this study.

## 5 CONCLUSIONS

Visit‐to‐visit BPV was an independent predictor of all‐cause mortality, when accounting for conventional risk factors or FRS. BPV combined with FRS conferred an increased risk for all‐cause mortality in the SPRINT trial, and the clinical significance of BPV should be further investigated.

## CONFLICT OF INTEREST

All authors declare no conflict of interest.

## AUTHOR CONTRIBUTIONS

Chang‐Sheng Sheng and Jingyan Tian had full access to all the data in the study and take responsibility for the accuracy of the data analysis, and participated in the study design and paper revision. Yi Cheng and Jian Li performed the studies and drafted the manuscript. Xinping Ren and Dan Wang helped with the statistical analysis. Yulin Yang and Ya Miao checked the accuracy of the analysis and involved in review the English language and grammar. All the authors read and approved the final manuscript.

## Supporting information

Supplementary information

## Data Availability

Our post hoc analysis used database of Systolic Blood Pressure Intervention Trial (SPRINT) from the National Heart, Lung and Blood Institute (NHLBI) Biologic Specimen and Data Repository Information Coordinating Centre (https://biolincc.nhlbi.nih.gov/studies/sprint/). The SPRINT data is a sharing recourse for scientific research and is available at NHLBI upon reasonable request, as per the Centre's data sharing philosophy.
