# Supplementary material for: Visit‐to‐visit office blood pressure variability combined with Framingham risk score to predict all‐cause mortality: A post hoc analysis of the systolic blood pressure intervention trial
Source: J Clin Hypertens (Greenwich). 2021 Jul 3;23(8):1516–25. doi: 10.1111/jch.14314 (PMC8678842; doi:10.1111/jch.14314)
Supplement: Supplementary file 1 — Supplementary information [file JCH-23-1516-s001.docx]

**Supplemental Material**

**Visit-to-Visit Office Blood Pressure Variability Combined with Framingham Risk Score to Predict All-cause Mortality**

A post hoc analysis of the Systolic Blood Pressure Intervention Trial (SPRINT)

Yi Cheng MD^*^, Jian Li MD^*^, Xinping Ren MD, PhD, Dan Wang MD, Yulin Yang MD, Ya Miao MD, Chang-Sheng Sheng MD PhD, Jingyan Tian MD PhD

**Table S1. SBP and DBP in two treatment groups over the follow-up**

|  | **Standard treatment** | | |  | **Intensive treatment** | | |
| --- | --- | --- | --- | --- | --- | --- | --- |
| **Follow-up** | No. of participants | SBP mean  (mmHg) | DBP mean  (mmHg) |  | No. of participants | SBP mean  (mmHg) | DBP mean  (mmHg) |
| baseline | 4673 | 139.7 ± 15.4 | 78.0 ± 12.0 |  | 4670 | 139.7 ± 15.8 | 78.2 ± 11.9 |
| 1M | 4500 | 133.2 ± 14.6 | 74.3 ± 11.4 |  | 4515 | 127.9 ± 15.1 | 71.9 ± 11.3 |
| 2M | 4378 | 133.3 ± 13.6 | 74.5 ± 11.3 |  | 4391 | 124.8 ± 14.0 | 70.1 ± 10.7 |
| **After selection** |  |  |  |  |  |  |  |
| 3M | 3995 | 133.6 ± 13.3 | 74.6 ± 11.1 |  | 4001 | 122.8 ± 13.4 | 69.0 ± 10.4 |
| 6M | 3995 | 134.5 ± 13.3 | 75.1 ± 11.3 |  | 4001 | 121.6 ± 13.0 | 68.5 ± 10.2 |
| 9M | 3995 | 135.0 ± 12.9 | 75.1 ± 11.2 |  | 4001 | 120.9 ± 13.1 | 67.8 ± 10.2 |
| 12M | 3995 | 136.2 ± 13.5 | 76.2 ± 11.1 |  | 4001 | 121.2 ± 13.4 | 68.5 ± 10.0 |
| 15M | 3850 | 134.7 ± 12.7 | 74.8 ± 11.3 |  | 3844 | 120.0 ± 12.6 | 67.3 ± 10.0 |
| 18M | 3797 | 135.0 ± 13.1 | 74.9 ± 11.3 |  | 3804 | 119.9 ± 12.5 | 67.1 ± 10.1 |
| 21M | 3733 | 135.2 ± 13.2 | 74.9 ± 11.4 |  | 3763 | 119.9 ± 12.7 | 67.0 ± 10.1 |
| 24M | 3701 | 136.8 ± 13.0 | 76.1 ± 10.8 |  | 3732 | 121.1 ± 13.8 | 67.9 ± 10.1 |
| 27M | 3661 | 135.0 ± 12.9 | 74.5 ± 11.2 |  | 3671 | 119.4 ± 12.8 | 66.5 ± 10.1 |
| 30M | 3518 | 135.1 ± 12.9 | 74.6 ± 11.2 |  | 3531 | 119.5 ± 12.7 | 66.7 ± 10.1 |
| 33M | 3113 | 135.2 ± 12.9 | 74.8 ± 11.1 |  | 3157 | 118.8 ± 12.4 | 66.5 ± 9.9 |
| 36M | 2608 | 135.9 ± 13.3 | 75.2 ± 11.1 |  | 2663 | 120.1 ± 13.4 | 67.0 ± 10.0 |
| 39M | 2075 | 134.8 ± 13.5 | 74.5 ± 11.3 |  | 2136 | 118.7 ± 12.8 | 66.1 ± 10.2 |
| 42M | 1561 | 135.4 ± 13.1 | 74.3 ± 10.9 |  | 1612 | 119.2 ± 13.5 | 66.3 ± 10.1 |
| 45M | 1149 | 134.6 ± 13.3 | 73.8 ± 11.3 |  | 1190 | 119.0 ± 12.7 | 66.2 ± 10.1 |
| 48M | 715 | 136.6 ± 13.5 | 75.1 ± 10.6 |  | 756 | 120.4 ± 14.0 | 66.8 ± 10.0 |
| 51M | 398 | 133.7 ± 13.0 | 72.6 ± 11.4 |  | 409 | 118.0 ± 11.5 | 65.5 ± 9.9 |
| 54M | 100 | 135.8 ± 13.1 | 73.6 ± 11.3 |  | 108 | 118.6 ± 13.1 | 64.4 ± 9.1 |

SBP, systolic blood pressure; DBP, diastolic blood pressure; M, month(s) since follow-up started. Values are mean ± SD.

**Table S2.** **Hazard ratio of SBP variabllity for all-cause mortality (five BP measurements)**

|  | Overall  (n=7694) | |  | Standard  (n=3850) | |  | Intensive  (n=3844) | |
| --- | --- | --- | --- | --- | --- | --- | --- | --- |
|  | Model 1 | Model 2 |  | Model 1 | Model 2 |  | Model 1 | Model 2 |
| **SBP VIM**  **Continuous** |  |  |  |  |  |  |  |  |
| +5.1 U | **1.27(1.13-1.43)¶** | **1.23(1.09-1.38)¶** |  | **1.30(1.13-1.50)¶** | **1.23(1.07-1.43)§** |  | **1.22(1.00-1.49)*** | 1.21(0.99-1.49) |
| **Quintiles** |  |  |  |  |  |  |  |  |
| Q1 | reference | reference |  | reference | reference |  | reference | reference |
| Q2 | **2.42(1.40-4.20)§** | **2.34(1.35-4.06)§** |  | **2.45(1.18-5.10)*** | **2.36(1.14-4.92)*** |  | **2.39(1.04-5.49)*** | 2.28(0.99-5.25) |
| Q3 | **2.05(1.17-3.60)*** | **1.96(1.11-3.44)§** |  | **2.14(1.02-4.50)*** | 2.01(0.96-4.23) |  | 1.95(0.82-4.64) | 1.91(0.80-4.56) |
| Q4 | **2.67(1.55-4.58)¶** | **2.50(1.45-4.29)¶** |  | **2.34(1.13-4.83)*** | **2.17(1.05-4.49)*** |  | **3.26(1.45-7.33)§** | **3.13(1.39-7.07)§** |
| Q5 | **2.99(1.76-5.08)¶** | **2.67(1.57-4.56)¶** |  | **3.20(1.60-6.39)¶** | **2.76(1.38-5.54)§** |  | **2.56(1.09-5.98)*** | **2.43(1.03-5.70)*** |
| **SBP MMD** |  |  |  |  |  |  |  |  |
| **Continuous** |  |  |  |  |  |  |  |  |
| +13.3mmHg | **1.26(1.13-1.41)¶** | **1.20(1.07-1.35)§** |  | **1.20(1.04-1.41)*** | 1.14(0.98-1.32) |  | **1.37(1.14-1.62)¶** | **1.32(1.10-1.58)§** |
| **Quintiles** |  |  |  |  |  |  |  |  |
| Q1 | reference | reference |  | reference | reference |  | reference | reference |
| Q2 | 1.07(0.62-1.83) | 1.03(0.60-1.77) |  | 1.22(0.63-2.37) | 1.19(0.61-2.30) |  | 0.75(0.29-1.98) | 0.72(0.28-1.91) |
| Q3 | **1.85(1.14-3.02)*** | **1.75(1.07-2.85)*** |  | 1.45(0.76-2.76) | 1.37(0.72-2.61) |  | **2.61(1.24-5.51)*** | **2.44(1.15-5.17)*** |
| Q4 | **1.92(1.19-3.10)§** | **1.81(1.12-2.93)*** |  | 1.79(0.97-3.29) | 1.71(0.93-3.14) |  | 2.13(0.98-4.62) | 2.03(0.93-4.42) |
| Q5 | **1.85(1.15-2.97)*** | **1.62(1.01-2.63)*** |  | 1.54(0.84-2.84) | 1.33(0.72-2.46) |  | **2.48(1.17-5.28)*** | **2.28(1.06-4.90)*** |

SBP, systolic blood pressure; VIM, variability independent of the mean; MMD, max-min difference; Q1-5, quintile 1-5.

Model 1 with adjustment of randomized group and FRS rank.

Model 2 further adjusted with history of CKD, glucose, mean number of antihypertensive agents, statin and aspirin use.

*P<0.05; §P<0.01; ¶P<0.001.

**Table S3.** **Hazard ratio of SBP VIM for all-cause mortality (six BP measurements)**

|  | Overall  (n=7445) | |  | Standard  (n=3721) | |  | Intensive  (n=3724) | |
| --- | --- | --- | --- | --- | --- | --- | --- | --- |
|  | Model 1 | Model 2 |  | Model 1 | Model 2 |  | Model 1 | Model 2 |
| **SBP VIM**  Continuous |  |  |  |  |  |  |  |  |
| +4.9 U | **1.28(1.13-1.45)¶** | **1.23(1.08-1.40)§** |  | **1.29(1.11-1.51)§** | **1.22(1.05-1.43)*** |  | **1.25(1.00-1.57)*** | 1.24(0.99-1.56) |
| Quintiles |  |  |  |  |  |  |  |  |
| Q1 | reference | reference |  | reference | reference |  | reference | reference |
| Q2 | **2.85(1.55-5.23)¶** | **2.74(1.49-5.03)§** |  | **2.34(1.04-5.29)*** | **2.30(1.02-5.19)*** |  | **3.76(1.51-9.38)§** | **3.64(1.45-9.15)§** |
| Q3 | **2.38(1.28-4.44)§** | **2.28(1.22-4.26)§** |  | **3.00(1.36-6.62)§** | **2.87(1.30-6.34)§** |  | 1.45(0.50-4.18) | 1.44(0.50-4.18) |
| Q4 | **2.64(1.43-4.86)§** | **2.46(1.33-4.53)§** |  | **2.32 (1.04-5.19)*** | 2.16(0.97-4.84) |  | **3.28(1.28-8.38)*** | **3.16(1.22-8.14)*** |
| Q5 | **3.08(1.70-5.60)¶** | **2.76(1.52-5.04)¶** |  | **3.10(1.44-6.70)§** | **2.66(1.23-5.77)*** |  | **3.07(1.18-8.01)*** | **3.02(1.15-7.96)*** |
| **SBP MMD** |  |  |  |  |  |  |  |  |
| **Continuous** |  |  |  |  |  |  |  |  |
| +13.5mmHg | **1.24(1.10-1.41)¶** | **1.18(1.04-1.34)§** |  | 1.16(0.99-1.37) | 1.10(0.93-1.29) |  | **1.40(1.15-1.68)¶** | **1.35(1.11-1.63)§** |
| **Quintiles** |  |  |  |  |  |  |  |  |
| Q1 | reference | reference |  | reference | reference |  | reference | reference |
| Q2 | 0.91(0.50-1.64) | 0.89(0.49-1.59) |  | 1.09(0.53-2.23) | 1.08(0.53-2.21) |  | 0.53(0.17-1.62) | 0.51(0.17-1.56) |
| Q3 | 1.51(0.88-2.60) | 1.45(0.84-2.50) |  | 1.48(0.74-2.95) | 1.45(0.73-2.91) |  | 1.59(0.66-3.84) | 1.51(0.62-3.66) |
| Q4 | **1.94(1.15-3.28)*** | **1.84(1.09-3.11)*** |  | 1.59(0.81-3.14) | 1.51(0.76-2.99) |  | **2.71(1.19-6.16)*** | **2.61(1.14-5.98)*** |
| Q5 | 1.60(0.95-2.71) | 1.42(0.83-2.40) |  | 1.31(0.67-2.57) | 1.14(0.58-2.25) |  | **2.31(1.00-5.31)*** | 2.12(0.91-4.94) |

SBP, systolic blood pressure; VIM, variability independent of the mean; MMD, max-min difference; Q1-5, quintile 1-5.

Model 1 with adjustment of randomized group and FRS rank.

Model 2 further adjusted with history of CKD, glucose, mean number of antihypertensive agents, statin and aspirin use.

*P<0.05; §P<0.01; ¶P<0.001

**Table S4.** **Hazard ratio of SBP variability for all-cause mortality in population without CVD (four BP measurements)**

|  | Overall  (n=6661) | |  | Standard  (n=3339) | |  | Intensive  (n=3322) | |
| --- | --- | --- | --- | --- | --- | --- | --- | --- |
|  | Model 1 | Model 2 |  | Model 1 | Model 2 |  | Model 1 | Model 2 |
| **SBP VIM**  Continuous |  |  |  |  |  |  |  |  |
| +5.6 U | **1.29(1.13-1.48)¶** | **1.25(1.09-1.44)§** |  | **1.33(1.12-1.57)§** | **1.28(1.08-1.51)§** |  | 1.23(0.97-1.56) | 1.21(0.94-1.55) |
| Quintiles |  |  |  |  |  |  |  |  |
| Q1 | reference | reference |  | reference | reference |  | reference | reference |
| Q2 | 1.15(0.64-2.08) | 1.10(0.61-1.99) |  | 1.50(0.69-3.28) | 1.43(0.66-3.11) |  | 0.80(0.32-2.03) | 0.78(0.31-1.97) |
| Q3 | **1.74(1.01-2.99)*** | 1.67(0.97-2.86) |  | 1.90(0.91-3.97) | 1.84(0.88-3.85) |  | 1.57(0.71-3.50) | 1.50(0.67-3.36) |
| Q4 | 1.65(0.96-2.85) | 1.56(0.91-2.70) |  | 1.77(0.85-3.67) | 1.69(0.81-3.51) |  | 1.56(0.68-3.56) | 1.47(0.64-3.37) |
| Q5 | **2.08(1.23-3.53)§** | **1.90(1.12-3.23)*** |  | **2.27(1.13-4.58)*** | **2.05(1.01-4.15)*** |  | 1.91(0.85-4.30) | 1.79(0.79-4.06) |
| **SBP MMD** |  |  |  |  |  |  |  |  |
| **Continuous** |  |  |  |  |  |  |  |  |
| +13.1mmHg | **1.31(1.15-1.49)¶** | **1.26(1.11-1.44)¶** |  | **1.28(1.09-1.53)§** | **1.23(1.04-1.46)*** |  | **1.35(1.10-1.65)§** | **1.32(1.07-1.63) *** |
| **Quintiles** |  |  |  |  |  |  |  |  |
| Q1 | reference | reference |  | reference | reference |  | reference | reference |
| Q2 | 1.44(0.76-2.74) | 1.41(0.74-2.67) |  | 1.63(0.69-3.84) | 1.55(0.66-3.67) |  | 1.25(0.48-3.29) | 1.26(0.48-3.31) |
| Q3 | **1.93(1.05-3.57)*** | 1.85(1.00-3.41) |  | 2.18(0.96-4.94) | 2.09(0.92-4.75) |  | 1.66(0.65-4.20) | 1.61(0.63-4.11) |
| Q4 | **2.27(1.26-4.09)§** | **2.11(1.17-3.81)*** |  | **2.40(1.09-5.27)*** | **2.24(1.02-4.92)*** |  | 2.17(0.89-5.29) | 2.05(0.84-5.03) |
| Q5 | **2.20(1.21-4.00)§** | **1.98(1.08-3.62)*** |  | 2.16(0.97-4.84) | 1.92(0.85-4.30) |  | 2.36(0.96-5.79) | 2.22(0.90-5.51) |

SBP, systolic blood pressure; VIM, variability independent of the mean; MMD, max-min difference; Q1-5, quintile 1-5.

Model 1 with adjustment of randomized group and FRS rank.

Model 2 further adjusted with history of CKD, glucose, mean number of antihypertensive agents, statin and aspirin use.

*P<0.05; §P<0.01; ¶P<0.001.

**Table S5. Cross-Tabulation of risk levels and variability tertiles in relation to all-cause mortality in population without CVD.**

|  | Low-risk | Intermediate-risk | High-risk |
| --- | --- | --- | --- |
| **VIM Tertiles**  Overall (n=6661) |  |  |  |
| T1 | Reference | 1.13(0.31-4.13) | 2.39(0.71-8.05) |
| T2 | 0.67(0.11-4.04) | 2.30(0.69-7.73) | **4.17(1.28-13.60)*** |
| T3 | 1.44(0.34-6.03) | 2.43(0.73-8.10) | **4.34(1.33-14.13)*** |
| Standard therapy (n=3322) | | | |
| T1 | Reference | 1.06(0.21-5.31) | 1.68(0.37-7.69) |
| T2 | 0.57(0.051-6.26) | 1.55(0.34-7.08) | **4.39(1.04-18.56)*** |
| T3 | 0.75(0.11-5.31) | 2.02(0.46-8.84) | 3.47(0.82-14.73) |
| Intensive therapy(n=3339) | | | |
| T1 | Reference | 1.32(0.15-11.89) | 3.95(0.50-30.92) |
| T2 | 0.91(0.057-14.62) | 4.02(0.52-31.31) | 3.57(0.45-28.35) |
| T3 | 3.23(0.33-31.24) | 3.35(0.42-26.93) | 6.35(0.83-48.86) |
| **MMD Tertiles**  Overall (n=6661) |  |  |  |
| T1 | Reference | 1.67(0.47-5.85) | 2.22(0.65-7.62) |
| T2 | 0.97(0.20-4.83) | 1.81(0.53-6.15) | **3.89(1.19-12.71)*** |
| T3 | 1.27(0.28-5.70) | 2.56(0.77-8.54) | **4.78(1.48-15.49)**§ |
| Standard therapy (n=3322) | | | |
| T1 | Reference | 1.50(0.32-7.08) | 1.28(0.26-6.15) |
| T2 | 0.47(0.042-5.16) | 0.90(0.19-4.33) | 3.85(0.91-16.31) |
| T3 | 0.75(0.11-5.34) | 2.10(0.48-9.16) | 3.58(0.85-15.13) |
| Intensive therapy(n=3339) | | | |
| T1 | Reference | 2.02(0.24-17.35) | 4.51(0.57-35.61) |
| T2 | 2.10(0.19-23.31) | 4.22(0.54-32.87) | 3.29(0.40-26.88) |
| T3 | 2.79(0.25-31.02) | 3.40(0.42-27.79) | **7.62(1.00-57.91)*** |

SBP, systolic blood pressure; VIM, variation independent of the mean; MMD, max-min difference; T1-3, tertile 1-3.

Models were adjusted with randomized group, history of CKD, glucose, mean number of antihypertensive agents, plus statin and aspirin use.

*P<0.05; §P<0.01.
